# Pilot study of lithium to restore intestinal barrier function in severe graft-versus-host disease

**DOI:** 10.1371/journal.pone.0183284

**Published:** 2017-08-17

**Authors:** Gideon Steinbach, David M. Hockenbery, Gerwin Huls, Terry Furlong, David Myerson, Keith R. Loeb, Jesse R. Fann, Christina Castilla-Llorente, George B. McDonald, Paul J. Martin

**Affiliations:** 1 Division of Clinical Research, Fred Hutchinson Cancer Research Center, Seattle, Washington, United States of America; 2 Department of Medicine, University of Washington, Seattle, Washington, United States of America; 3 Department of Haematology, Radboud University Medical Center, Nijmegen, The Netherlands; 4 Department of Pathology, University of Washington, Seattle, Washington, United States of America; 5 Department of Psychiatry, University of Washington, Seattle, Washington, United States of America; Jackson Laboratory, UNITED STATES

## Abstract

Severe intestinal graft-vs-host disease (GVHD) after allogeneic hematopoietic cell transplantation (HCT) causes mucosal ulceration and induces innate and adaptive immune responses that amplify and perpetuate GVHD and the associated barrier dysfunction. Pharmacological agents to target mucosal barrier dysfunction in GVHD are needed. We hypothesized that induction of Wnt signaling by lithium, an inhibitor of glycogen synthase kinase (GSK3), would potentiate intestinal crypt proliferation and mucosal repair and that inhibition of GSK3 in inflammatory cells would attenuate the deregulated inflammatory response to mucosal injury. We conducted an observational pilot study to provide data for the potential design of a randomized study of lithium. Twenty patients with steroid refractory intestinal GVHD meeting enrollment criteria were given oral lithium carbonate. GVHD was otherwise treated per current practice, including 2 mg/kg per day of prednisone equivalent. Seventeen patients had extensive mucosal denudation (extreme endoscopic grade 3) in the duodenum or colon. We observed that 8 of 12 patients (67%) had a complete remission (CR) of GVHD and survived more than 1 year (median 5 years) when lithium administration was started promptly within 3 days of endoscopic diagnosis of denuded mucosa. When lithium was started promptly and less than 7 days from salvage therapy for refractory GVHD, 8 of 10 patients (80%) had a CR and survived more than 1 year. In perspective, a review of 1447 consecutive adult HCT patients in the preceding 6 years at our cancer center showed 0% one-year survival in 27 patients with stage 3–4 intestinal GVHD and grade 3 endoscopic appearance in the duodenum or colon. Toxicities included fatigue, somnolence, confusion or blunted affect in 50% of the patients. The favorable outcomes in patients who received prompt lithium therapy appear to support the future conduct of a randomized study of lithium for management of severe GVHD with extensive mucosal injury.

**Trial Registration:** ClinicalTrials.gov NCT00408681

## Introduction

Acute graft-versus-host disease (GVHD), driven by alloreactive donor T cells, remains a significant complication of allogeneic hematopoietic cell transplantation (HCT). Among acute GVHD targets, intestinal involvement is the main source of morbidity and mortality [1]. The histological hallmark of intestinal GVHD is apoptosis of epithelial cells at the crypt base and secondary dropout of cells and crypts [2,3]. At the extreme severity GVHD results in progressive crypt loss and mucosal denudation of large segments of the intestine [4–6]. In our experience, mucosal recovery and survival at this stage are distinctly rare outcomes [1,5,7,8].

Steroid refractory intestinal GVHD is increasingly viewed as resembling a barrier dysfunction disorder characterized by an anatomic or physiologic barrier defect and a dysregulated immune response, resulting in a self-perpetuating cycle of chronic inflammation and barrier disruption [9–12]. In GVHD, the underlying defect is the immune mismatch between recipient and donor [4]. Mucosal injury results in exposure to luminal microbiota and damage associated molecular patterns that induce an inflammatory and cytokine cascade mediated by pattern recognition receptors and antigen presenting cells [9,13–18]. Aberrant toll-like receptor signaling, including a proinflammatory response to commensal bacteria, as well as dysregulated mucosal repair and dysbiosis contribute to this process [11,13,19–21]. The mucosal inflammation amplifies and perpetuates systemic GVHD and sustains the targeting of intestinal epithelium by effector immune cells and cytokines [22,9]. The clinical challenge is to support the immunosuppressed patient with a denuded mucosal barrier until adequate mucosal regeneration and resolution of mucosal inflammation have occurred. Current treatment of severe GVHD, utilizing broad spectrum immunosuppression, targets systemic GVHD but generally fails to resolve the mucosal pathology. Therapeutic agents to induce mucosal regeneration and specifically target the mucosal inflammatory cycle are lacking.

The study of lithium in patients with severe GVHD and mucosal denudation was based on the hypotheses that induction of Wnt signaling by lithium would potentiate intestinal crypt proliferation and mucosal healing and that inhibition of GSK3 in inflammatory cells would modulate the proinflammatory response to mucosal barrier dysfunction. Landmark studies have established that Wnt signaling, including the downstream β-catenin/Tcf4-mediated transcription, is essential for epithelial stem cell replication, crypt genesis and crypt proliferation [23,24]. Inhibition of Wnt, β-catenin or Tcf4 in animal models results in loss of intestinal crypts and in mucosal denudation [24–27]. Conversely, induction of Wnt signaling in vitro and in animal models results in proliferative epithelium [28,29]. In addition, it has been shown by Hans Clevers’ group that self-renewing intestinal crypt organoids can be generated in vitro by induction of Wnt signaling in isolated intestinal stem cells [30]. Induction of Wnt signaling by the recombinant Wnt agonist R-spondin1 has also been shown to repair radiation-induced intestinal mucosal injury and thereby suppress systemic GVHD in a rat model of HCT [31]. These observations demonstrated that systemic GVHD can be resolved by anatomic repair of mucosal barrier disruption, and that mucosal repair can be enhanced by induction of Wnt signaling.

Lithium is an inhibitor of glycogen synthase kinase 3 (GSK3), a constitutively active protein kinase with diverse substrates, which is also the key regulator of cytosolic β-catenin [32]. Inhibition of GSK3 by lithium promotes β-catenin/Tcf4-mediated transcription, the nuclear effector pathway of Wnt signaling [33,34] GSK3 inhibition was also shown to expand the crypt epithelial stem cell niche in vitro by modulating paneth cell and stem cell signaling [29,35]

In addition to potentiating Wnt signaling, lithium has antiinflammatory effects in experimental settings attributable to inhibition of GSK3 [36–40]. A large body of data indicates a role for GSK3 in modulating innate immune responses, including promotion of proinflammatory nuclear factor kappa-B (NF-κB)-mediated signaling [41–43]. Physiologically phosphorylated (kinase-inactive) GSK3 and experimentally inhibited GSK3 can induce the association of cAMP response element–binding protein (CREB) to the nuclear coactivator CREB–binding protein (CBP) and decrease the association of NF-κB p65 with CBP, thereby augmenting IL-10 production while reducing proinflammatory cytokine expression [36,43–46]. Inhibition of GSK3 can modulate dysregulated toll-like receptor signaling, as in disrupted, inflamed mucosa, to restore anti-inflammatory cytokine response [37,45,46]. Lithium and other GSK3 inhibitors had protective effects in experimental animal models of enteritis and colitis [36,46–49]. attributed to both anatomic tissue repair and to antiinflammatory effects. Recently, GSK3 inhibition was also shown to prevent lethal GVHD in a humanized xenograft model in mice [50].

Our observational study of lithium explored a new approach to the treatment of severe GVHD focused on restoration of mucosal barrier function. As such, it was intended to provide preliminary data to guide the design of a randomized clinical trial of lithium for potential stimulation of intestinal mucosal recovery and resolution of mucosal inflammation in patients with severe intestinal GVHD and extensively denuded intestinal mucosa.

## Patients and methods

### Study design

This was an open-label, single arm, observational pilot study conducted among allogeneic HCT recipients ≥ 18 years of age and aimed to provide preliminary observations to potentially guide the design of a structured, randomized clinical trial of lithium. Patients were enrolled between July 18 2006 and August 31, 2010, and followed to May 1, 2015, or to date of death, if earlier. All aspects of the study, including recruitment, monitoring and data collection were conducted at the Fred Hutchinson Cancer Research Center (FHCRC) outpatient and hospital facilities (the Seattle Cancer Care Alliance and University of Washington Medical Center in Seattle, Washington). The study was approved by the FHCRC Institutional Review Board (Protocol 2080.00, June 28, 2006). The clinical investigation was conducted according to the principles expressed in the Declaration of Helsinki. Written informed consent was obtained from all the participants. The study was submitted to NCI’s Physician Data Query (PDQ) for registration on August 11, 2006 after we established that patient recruitment to the pilot study was feasible, and was registered by PDQ at www.clinicaltrials.gov, # NCT00408681, on December 6, 2006. There are no ongoing or related trials of Lithium in patients with GVHD at the FHCRC.

The protocol for this trial and supporting TREND checklist are available as Supporting Information (S1 Checklist and S2 Protocol).

### Eligibility and recruitment

Patients were eligible for inclusion in the study if they had a) denuded intestinal mucosa caused by GVHD, defined as ulceration or sloughing of the epithelium in at least one third of the surface area in a 30 cm colonic segment (i.e., rectosigmoid, descending or transverse colon) or in at least one fifth of the surface area of the second portion of the duodenum, as estimated by endoscopic evaluation and imaging, or b) a clinical diagnosis of persistent severe intestinal GVHD after initial treatment with glucocorticoids for at least 7 days as measured by stool volumes > 500 mL per day or persistent hemorrhage detectable by visual inspection of the stool.

Patients were ineligible for the study if they had significant renal dysfunction (estimated GFR < 30 mL/min), persistent or recurrent malignancy, presence of a primary cause of intestinal symptoms or ulceration other than GVHD, or any psychological condition potentially limiting treatment or compliance.

Patients with denuded intestinal mucosa were identified by the FHCRC endoscopists (GS, DH, GBM) and the transplant teams and referred to the study coordinator for evaluation. A rigorous attempt was made to identify and refer all patients with denuded mucosa who presented during the study period by monitoring all endoscopies in this period. In addition, a few patients with severe intestinal GVHD were selected by the transplant teams and referred for evaluation by the study coordinator. Referred patients who met eligibility criteria and provided consent were enrolled in the study. All patients identified to have denuded mucosa in our institution during the study period who met eligibility criteria and consented to the study were enrolled and evaluated for outcome. One thousand seven hundred and twelve adult allogeneic HCT were performed at the FHCRC during the study period. All transplant patients are closely monitored for outcome by the FHCRC teams.

**Fig 1:**
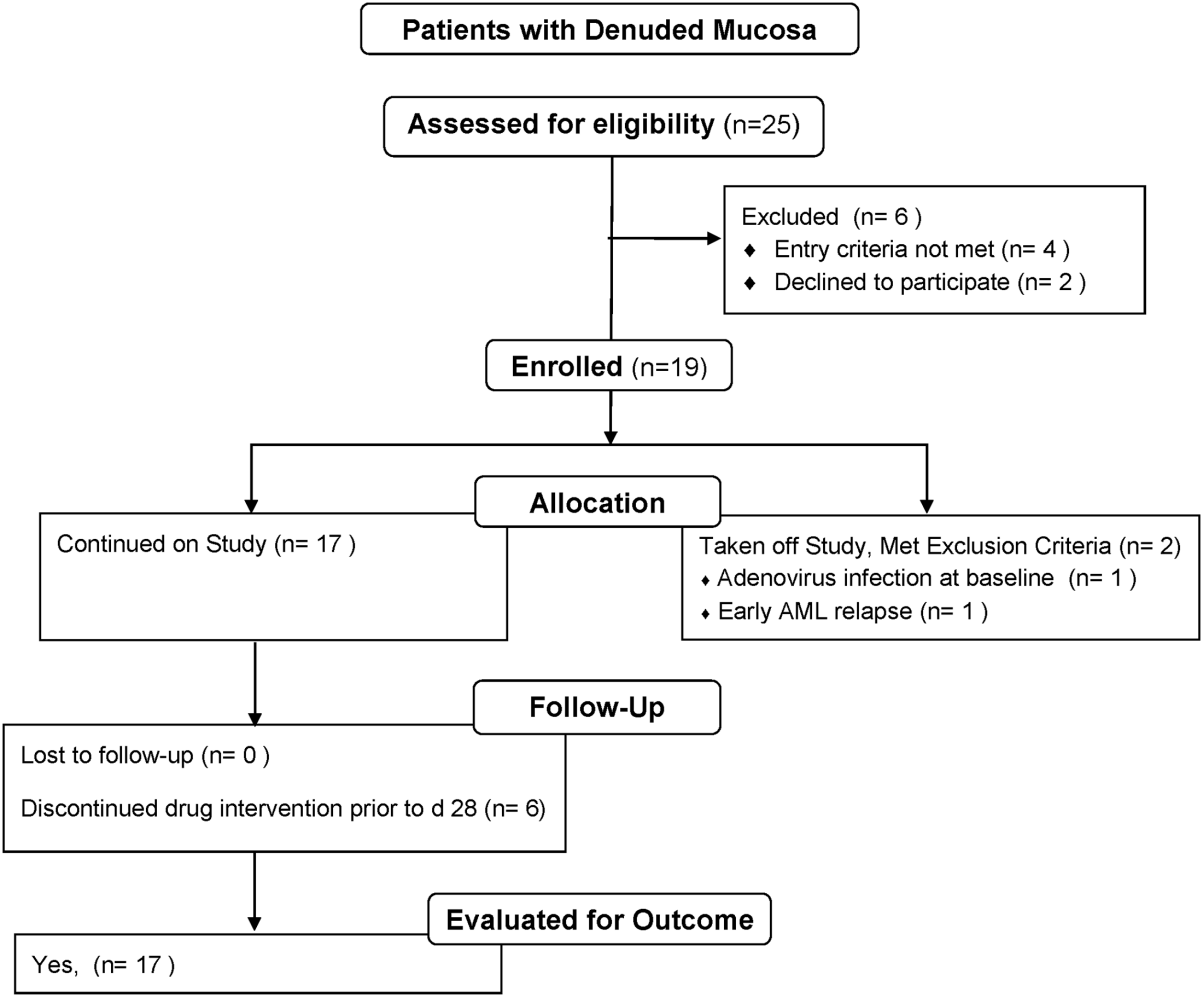
Flow chart of patients with denuded intestinal mucosa.

Fig 1 illustrates the patient flow for patients with denuded mucosa. Twenty five patients with denuded intestinal mucosa were identified and referred to the study. Six were excluded, of whom 4 did not meet eligibility criteria and 2 refused consent. Three of the six also chose hospice care. Two patients were taken off study due to diagnosis of adenovirus infection on baseline endoscopy in one patient, and early relapse of AML requiring chemotherapy in another. The latter patient is included in the drug safety reporting.

Five patients without denuded mucosa were selected by the transplant teams based on the impression of severe GVHD and referred for study enrollment. Following enrollment, one patient was retrospectively noted to not have met study eligibility criteria and was not evaluated for outcome measures other than safety reporting. This patient had intact mucosa and mild GVHD responsive to low dose prednisone at 1 mg/kg/d, which was started after the baseline endoscopy. Another patient was diagnosed with adenovirus infection at baseline, a study exclusion criterion, and taken off study. Three patients with intact mucosa were eligible for outcome evaluation. The large majority of patients with severe GVHD and intact mucosa seen at our institution during the study period were not referred to the study. S1 Fig illustrates the patient flow for patients with intact intestinal mucosa.

### Administration of lithium

In addition to treatment of GVHD, study patients received extended-release lithium carbonate tablets (450 mg) starting at 450 mg orally per day. Initially the dose was adjusted, up to 3 doses daily, to maintain the serum lithium trough concentration at 0.8 to 1.2 mmol/L. Subsequently, the dose was restricted to once or twice daily and adjusted to maintain serum concentrations of 0.5 to 1.0 mmol/L after we observed dulled affect at higher concentrations. Serum lithium concentrations were monitored beginning at one week after the first dose. Lithium was administered by the nursing staff in the hospital setting for the full duration in 9 patients and for the initial 12–49 days in 11 patients. Medication was self-administered in the outpatient setting.

The timing of initiation and the duration of lithium administration were at the discretion of the patient care team and were not dictated by the study protocol. The duration of lithium administration was limited to 8 weeks, with the option to extend administration outside the auspices of the protocol. Cessation of lithium administration was required in the event of significant toxicity, including renal dysfunction (estimated GFR < 30 mL/min) or recurrent or secondary malignancy.

### Prophylaxis and treatment of GVHD

Individualized GVHD therapy was prescribed by the patient care team. Current practice at the time of the study included GVHD prophylaxis with a calcineurin inhibitor (cyclosporine or tacrolimus) in combination with either methotrexate or mycophenolate mofetil (MMF) after myeloablative conditioning. A calcineurin inhibitor in combination with MMF was used after reduced intensity conditioning. Patients with HLA-haploidentical donors also received high-dose cyclophosphamide after HCT. Initial treatment of acute GVHD generally consisted of corticosteroids at a prednisone-equivalent dose of 1–2 mg/kg/day, depending on the severity, and continued administration of a calcineurin inhibitor. Most patients also received oral beclomethasone dipropionate in corn oil with or without oral enteric-coated budesonide. Other immunosuppressive agents were added at the discretion of the patient care team if patients had an unsatisfactory response or rapid progression of severe symptoms. MMF (or enteric coated mycophenolic acid) and sirolimus were often used in this setting. Agents with a high toxicity profile or poorly defined efficacy profile were reserved for severe GVHD considered to be refractory or unlikely to respond to standard measures. For the purpose of the study reporting, these agents are designated as “salvage therapy” and include infliximab, etanercept, rabbit or horse antithymocyte globulin, alemtuzumab, pentostatin, extracorporeal photopheresis, and a clinical trial of mesenchymal stem cells versus placebo.

Patients were considered to have corticosteroid-refractory GVHD if they experienced 3 days with progressive manifestations of GVHD, 1 week with persistent, unimproving grade 3 GVHD or inability to taper steroids after 2 weeks, during treatment at prednisone-equivalent dose of 2 mg/kg or higher. Patients treated with salvage therapy in addition to steroids at 2 mg/kg were also considered to have corticosteroid-refractory GVHD.

### Evaluations

Study evaluations included patient interviews and review of the patient’s clinical course and laboratory, histologic and endoscopic findings. Endoscopic evaluations were performed only as clinically indicated and were not prescribed by the study protocol. Medical records were reviewed by study personnel to monitor for adverse events.

### End points

The primary endpoints, in addition to safety, were the clinical response of GVHD among all patients and the mucosal response in the subset of patients with endoscopically documented denuded intestinal mucosa, among patients who received lithium for at least 28 days. Assessment of survival and informal comparison to historical data was also planned.

### Criteria for clinical response

Complete response (CR) of intestinal GVHD was defined as the absence of any symptoms referable to intestinal GVHD. Partial response (PR) was defined as clearing of abdominal pain (or withdrawal of opioid analgesic requirements in patients treated for abdominal pain) and of grossly visible bleeding if present, and resolution of diarrhea or decrease in the three day average stool volume by ≥ 500 mL in patients with stool volumes of ≥ 500 mL. Progression of GVHD was defined as an increase in the three day average stool volume by > 500 mL, or the development of new abdominal pain (or new opioid analgesic requirements) or new intestinal bleeding.

### Criteria for mucosal response

Complete mucosal recovery was defined as the appearance of intact mucosa with at least 98% of the luminal surface covered by epithelium. Partial response was defined as the appearance of mostly intact mucosa with at least 80% improvement or less than 10% denuded. Limited response was retrospectively designated to describe visible improvement that was less than partial response. Failure to respond was defined as failure to meet partial response criteria within 60 days.

### Termination of the study

Termination of the study was required if none of the initial 6 patients showed complete or partial functional or mucosal anatomic interim recovery at 4 weeks of lithium administration. Continuation of the study was also subject to institutional review board monitoring of adverse events.

### Timing of events

The timing of the baseline endoscopy diagnostic of denuded mucosa, or baseline endoscopy prior to enrollment in patients without denuded mucosa, is indicated as days after HCT. The onset of lithium administration is indicated as days after baseline endoscopy. The timing of subsequent events, including GVHD response and survival, is indicated as days after the onset of lithium administration.

### Statistical plan

The statistical plan called for a descriptive evaluation of the findings, since the trial had an open label pilot design in which the timing of initiation of lithium therapy, the duration of therapy and the frequency and timing of endoscopic reexaminations were at the clinical discretion of the patient care team and were not prescribed by the protocol.

Comparison to historical results: The statistical considerations called for an assessment of survival and indicated that historical survival results would be used informally as a basis for judging the merits of more formal studies to evaluate the efficacy of lithium. To that end, the data from two large FHCRC cohorts reported by Gooley et al [51] and Castilla-Llorente et al [1] are retrospectively cited in the discussion section. Gooley et al reviewed the overall mortality in 1148 consecutive transplants at the FHCRC from 2003 to 2007. Castilla-Llorente et al reviewed the incidence and associated mortality of stage 3–4 intestinal GVHD among 1465 consecutive transplants (1447 adults) at the FHCRC in the 6 years from 2000 to 2005.

## Results

### Patient characteristics

Patient characteristics are presented in Table 1. There were 13 women and 7 men of median age 49.5 years (range 22–63 years) who were enrolled and eligible for evaluation of outcome. The median time from HCT to enrollment was 90 days (range 22–800 days). The patients were treated with corticosteroids at a prednisone- equivalent dose of 2 mg per kilogram per day on or before the first day of lithium (n = 18) or within 4 days afterwards (n = 2). Sixteen had steroid-refractory GVHD at the onset of lithium administration, and 4 developed steroid-refractory GVHD during follow-up. Seventeen of the 20 patients had endoscopically documented denuded mucosa in the duodenum alone (n = 7), the colon alone (n = 7) or both (n = 3). Three patients had intact mucosa.

**Table 1:** Patient characteristics.

Patient	Diagnosis	HLA Matching	Myeloablative conditioning regimen	Original GVHD prophylaxis	lithium start day after HCT	Site denuded	
						D	C
1	NHL	M	N	CSP/MMF	34	Y	Y
2	ALL	MM	Y	Tac/MTX	45	N	Y
3	AML	M	Y	Tac/MTX	133	N	Y
4	NHL	M	N	Tac/MMF	105	N	Y
5	AML	MM (CB)	Y	CSP/MMF	48	Y	N
6	AML	M	Y	Tac/MTX	215	Y	N
7	MM	H	N	Cy/Tac/MMF	110	N	Y
8	CML	M	N	CSP/MMF	138	N	Y
9	MM	MM	N	CSP/MMF	140	N	Y
10	AML	MM	N	CSP/MMF	54	Y	N
11	NHL	MM	N	CSP/MMF	32	Y	Y
12	ALL	MM	Y	Tac/MTX	151	N	Y
13	ALL	MM	Y	Tac/MTX	40	Y	N
14	MDS	M	Y	Tac/MTX	103	Y	Y
15	CML	M	Y	Tac/MTX	77	Y	N
16	AML	MM	Y	Tac/MTX	22	Y	N
17	AML	MM	Y	Tac/MTX	22	Y	N
18	AA	M	Y	CSP/MMF	42	N	N
19	PNH	M	Y	Tac/MTX	134	N	N
20	CLL	MM	N	CSP/MMF	49	N	N

D, indicates duodenum; C, colon; AA, aplastic anemia; ALL, acute lymphoid leukemia; AML, acute myeloid leukemia; CB, cord blood; CLL, chronic lymphocytic leukemia; CML, chronic myeloid leukemia; MDS, myelodysplastic syndrome; MF, myelofibrosis; MM, multiple myeloma; NHL, non-Hodgkin lymphoma; PNH, paroxysmal nocturnal hemoglobinuria; HLA: M, matched; MM, mismatched; H, haploidentical. CSP, cyclosporine; Cy, cyclophosphamide; MTX, methotrexate; and Tac, tacrolimus; Y, yes; N, no.

### Response after administration of lithium

Table 2 summarizes the systemic agents given for prevention or treatment of GVHD. Ten of the 20 patients, 50%, had a CR of intestinal GVHD, of whom 8 survived >1 year (median 5 years, range 1.9 to >7 years) after the onset of lithium administration (Table 3). Ten patients who did not have CR died with severe GVHD within 3 months. Baseline parameters that differed between patients who did and did not achieve a sustained CR and survival past 1 year included the interval time between the onset of lithium administration and the baseline endoscopy (a treatment variable at baseline), and the timing of initiation of salvage therapy for GVHD before the onset of lithium administration (a presumed measure of the duration of refractory GVHD at baseline).

**Table 2:** Systemic agents given for the prevention or treatment of GVHD.

Patient	GVHD prophylaxis at start of lithium	GVHD treatment in addition to Prednisone, 2mg/kg/d* (days before -, or after, lithium start)					Number of salvage agents given ≥7 days before lithium
1	CSP	MTX (1)	*Pento (38)*				0
2	Tac	*rATG (0)*					0
3	Tac	MMF (-8)	*Inflix (2)*	Sirol (45)			0
4	Tac/MMF	MMF (-17)^‡^	*rATG (1)*	Sirol (74)^†^			0
5	MMF	Tac (-21)^†^	*rATG (12)*	Sirol (47)			0
6	None	Tac (-32)	MMF (0)				0
7	Tac/MMF	*rATG (4)*					0
8	CSP	MMF (-19)	CSP (-11)^‡^	*Pento (0)*			0
9	CSP	MTX (–40)	MMF (-40)	Tac (13)	*Etan (19)*	*Inflix (80)*	0
10	CSP/MMF	*hATG (-14)*	*MSC (-14)*	*Inflix (14)*	Tac (15)	Sirol (16)	2
11	CSP/MMF	*rATG (-7)*	*Inflix (2)*	Sirol (10)			1
12	Tac	*rATG (-3)*	MMF (12)				0
13	Tac	*Inflix (-7)*	*Pento (0)*	*rATG (9)*	MMF (30)^†^	Sirol (30)^†^	1
14	Tac	*rATG (2)*	*Inflix (17)*				0
15	None	Tac (-27)	*Inflix (-20)*	*ECP (-7)*	MMF (23)		2
16	Tac	*Alemt (-1)*	MMF (8)				0
17	MMF	*rATG (5)*	*Alemt (7)*^¶^				0
18	CSP/MMF	*rATG (-8)*	*Inflix (5)*				1
19	None	*Inflix (-40)*	MMF (-34)	*ECP (-17)*	Sirol (-17)	*rATG (11)*	2
20	CSP/MMF	*rATG (-9)*					1

Alemt indicates alemtuzumab; Etan, etanercept; Inflix, infliximab; MSC, mesenchymal stem cell versus placebo study; and Pento, pentostatin. “Salvage therapy” agents are designated in italics (see Methods).

^†^added as substitute for another drug;

^‡^was on taper, dose increased.

^¶^alemtuzumab substituted for ATG due to ATG toxicity.

**Table 3:** Outcomes after lithium administration.

Patient	lithium start day after endoscopy	lithium administration duration, days	Intestinal GVHD Response	Survival (S) at 2 years or day expired*	Causes of death
1	0	56	CR	S	
2	0	43	CR	S	
3	1	57	CR	S	Relapse (1730^†^) 1767*
4	2	44	CR	S	Relapse (611^†^, 2151^†^) 2181*
5	3	108	CR	S	
6	3	40	CR	S	
7	3	30	CR	S	
8	1	19	CR	714	cGVHD, BOS
9	7	172^‡^	CR	302	BOS, cGVHD
10	0	25	NR	39	GVHD, CMV, Infection
11	5	31	NR	33	MOF, GVHD, Infection
12	6	19	PR	58	Liver GVHD, Infection
13	0	7	NR	65	DAH, GVHD, Infection
14	9	34	NR	63	GVHD, Infection
15	22	56	NR	85	GVHD
16	2	8	NR	27	HUS, GVHD, MOF
17	3	8	NR	41	GVHD
18	10	8	NR	11	GVHD
19	11	13	NR	24	GVHD, CMV
20	10	18	CR	210	Infection, GVHD

NR indicates no response; ARDS, adult respiratory distress syndrome; BOS, bronchiolitis obliterans syndrome; DAH, diffuse alveolar hemorrhage; HUS, hemolytic uremic syndrome; cGVHD, chronic GVHD; CMV, cytomegalovirus; and MOF, multiorgan failure.

*day expired.

^†^day of recurrent malignancy after start of lithium.

^‡^lithium withheld day 8–19

In 12 patients, lithium administration was started within 3 days of the baseline endoscopy and the diagnosis of denuded mucosa. Eight of these 12 patients, 67%, had a CR and survived for more than 1 year. Among the patients who started Li more than 3 days after the baseline endoscopy, 2 had a CR, but none survived 1 year.

In 7 patients, treatment of refractory GVHD with salvage therapy was started 7 or more days (range 7–40 days) before the onset of lithium administration. One of these 7 patients had a CR, but none survived 1 year. Use of salvage therapy, lacking established efficacy and safety profile, was reserved for patients with steroid refractory GVHD deemed unlikely to respond to continued standard treatment. This subset of 7 patients had persistent refractory disease during salvage therapy before the onset of lithium administration. In contrast, none of the 8 patients who survived more than 1 year had been treated with salvage therapy before the onset of lithium administration (Table 4).

**Table 4:** Characteristics of 1-year survivors and non-survivors.

Characteristic	Survivors (N = 8)	Nonsurvivors (N = 12)
lithium started ≤ 3 days after endoscopic diagnosis of denuded mucosa, N (%)	8 (100)	4 (33)
Salvage therapy for refractory GVHD ≥ 7 days before lithium, N (%)	0 (0)	7 (58)
lithium started ≤ 3 days after endoscopy and no salvage therapy for GVHD ≥ 7 days before lithium, N (%)	8 (100)	2 (17)
lithium started ≤ 3 days after endoscopy and given for ≥ 19 days, N (%)	8 (100)	1 (8)

### Duration of lithium administration

Lithium was administered for a median of 30 days (range 7–172 days). Only 11 patients took lithium for the planned 28 day minimum duration of administration (median duration, 44 days). Among the other 9 patients, administration of lithium was discontinued before day 28 due to toxicity or perceived inefficacy. Among the 3 patients who died prior to day 28, administration of lithium had already been discontinued 3, 11 and 19 days earlier. There were no 1 year survivors among patients who took lithium for less than 19 days. Seven of the eight 1 year survivors took lithium for 30 or more days.

### Mucosal recovery in patients with documented denuded intestinal mucosa (Table 5)

**Table 5:** Mucosal response among patients with denudation*.

Response^†^	Week after the onset of lithium administration				
	2–3	4	5	6–7	9–11
Number of patients examined	8 (6D, 5C)	2 (1D, 1C)	1 (D)	3 (1D, 3C)	4 (3C, 2D)
No response, N (sites)	3 (3D, 1C)				
Regenerative changes, N (sites)	3 (2D, 2C)				
Limited response, N (sites)		1 (C)			
Partial response, N (sites)	2 (1D, 2C)	1 (D)	1 (D)		2 (C)
Complete response, N (sites)				3 (1D, 3C)	2 (2D, 1C)

D, duodenum; C, colon.

*3 patients with denuded mucosa did not have a follow-up endoscopic evaluation.

^†^A total of 18 endoscopies (esophagogastroduodenoscopy, sigmoidoscopy/partial colonoscopy, or both) were performed in 14 patients. No response, no change; regenerative changes, diffuse foci of regenerative mucosa; limited response (LR), clearly visible improvement, less than PR; partial response (PR), more than 80% improvement; complete response (CR), more than 99% intact at 1 exam on week 6, normal in 4. Three patients had serial follow-up exams: 1) PR week 2, CR with abnormal mucosa week 6, CR week 10; 2) LR week 4, PR week 11; 3) PR week 5, CR week 11.

**Fig 2:**
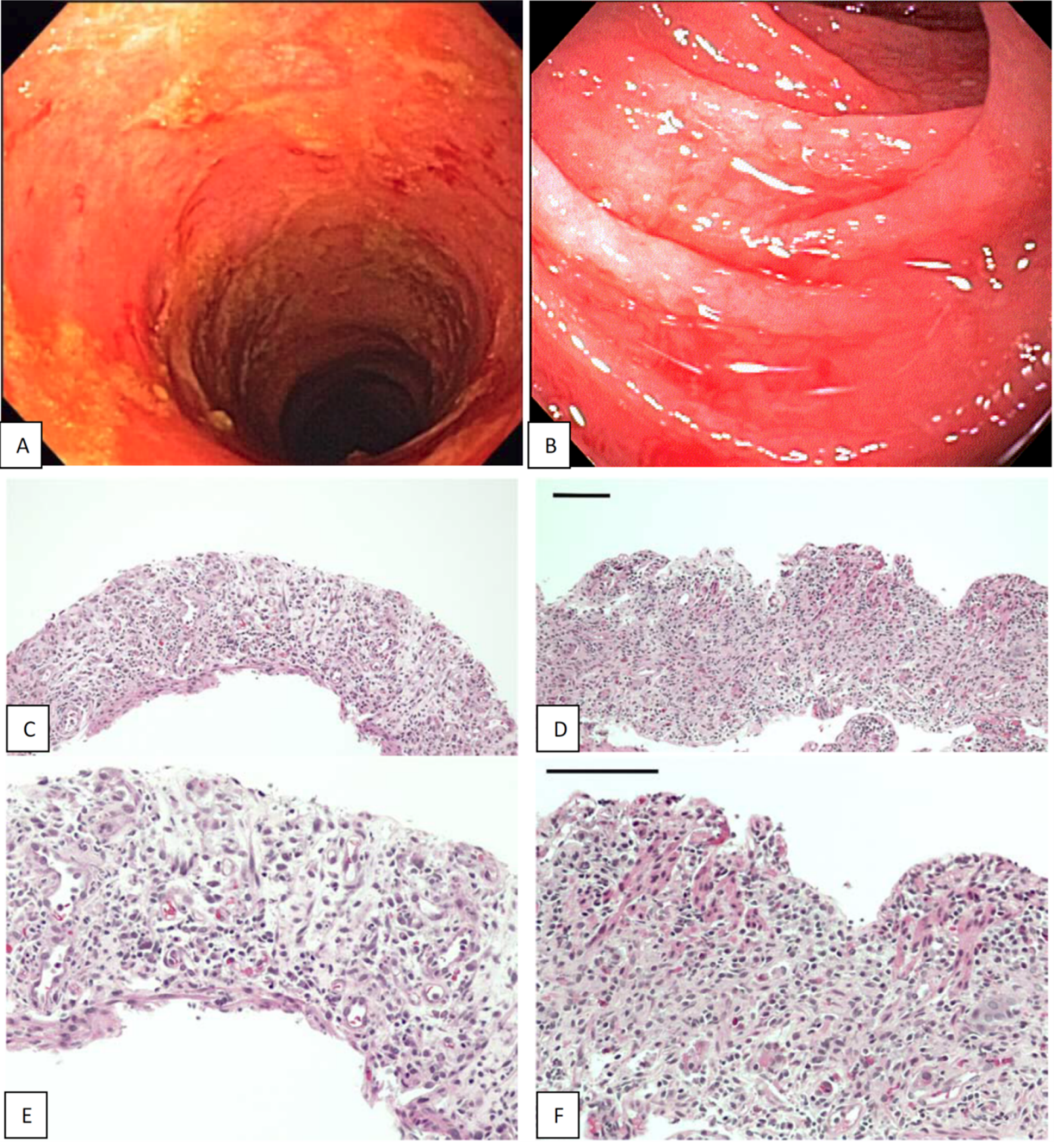
Endoscopic appearance (A, B) and histology (C-F) of denuded mucosa in the colon (left) and duodenum (right) at baseline in two patients who experienced CR of GVHD and survived more than one year. Surface epithelium and crypts are virtually absent in the colon and duodenum and villi are absent in the duodenum (C,D H&E x10; E,F H&E x20; bar size = 100uM).

The estimated extent of mucosal denudation at baseline was similar in patients who survived more than one year and in those who did not. Specifically, very extensive mucosal denudation, far exceeding entry criteria, was seen at baseline in 7 of the 8 patients who survived more than 1 year, with almost complete denudation of the visualized second and third portion of the duodenum in 2 patients and extensive denudation of most of the visualized colon (sigmoid, descending and transverse) in 6 patients (Fig 2). Follow-up endoscopic evaluations were limited because patients were examined endoscopically only for clinical indications. The earliest mucosal PR was observed during week 2 after the onset of lithium administration, and the earliest CR was observed during week 6. Five of the 6 patients with mucosal PR survived for at least 9 months. All 4 patients with mucosal CR (including 3 with a prior PR) survived more than a year. CR is assumed in the 4 long-term survivors who did not have a follow-up endoscopic evaluation during the study period.

### Adverse events

Lithium trough serum concentrations remained below 1.0 mEq/L (therapeutic range 0.5 to 1.50 mEq/L) in all but 2 patients who reached maximal concentrations of 1.04 and 1.27 mEq/L. Patients commonly experienced some degree of fatigue, decreased level of attention, somnolence or flattened affect (Table 6). The changes were considered to be related to lithium, but were likely multifactorial in at least six of the patients. Two patients experienced multifactorial severe somnolence during intensive care. Mild intermittent tremor, possibly related to lithium, was seen in one patient with baseline intermittent tremor attributed to tacrolimus. All patients received medications with overlapping toxicity profiles. Common concomitant medications included opioids, benzodiazepines, phenothiazines, calcineurin inhibitors. Administration of lithium was discontinued due to elevated liver function tests and skin blistering in two patients with documented severe GVHD of the liver and skin, respectively. Other causes for discontinuing lithium administration included polyuria (in a patient with preceding polyuria) and leukocytosis, both of which were multifactorial in origin. No grade 3 signs or symptoms were considered to be exclusively attributable to lithium. Three patients (15%) had recurrent malignancy. One had leukemia on day 5 after the onset of lithium administration and was taken off study (but is included in the toxicity monitoring, Fig 1). A second patient had recurrent extranodal diffuse large B cell lymphoma on day 611 and again on day 2151. The third patient had recurrent AML with a chloroma on day 1730.

**Table 6:** Adverse events*.

Event	N (%)
Mental status changes	10 (45)
Somnolence, fatigue	6 (27)
Confusion	3 (14)
Apathy	2 (9)
Polyuria	1 (5)
Leukocytosis	1 (5)
Nausea/vomiting	2 (9)
Reasons for discontinuation of Li^+^	
Mental status changes	7 (32)
Response	7 (32)
Lack of response	2 (9)
Blistering of skin	1 (5)
Elevated liver function tests	1 (5)
Leukocytosis	1 (5)
Polyuria	1 (5)
Low Li^+^ serum levels	1 (5)
Change to comfort care	1 (5)

*Adverse events observed in patients who received lithium carbonate. No grade 3 toxicity was attributable exclusively to lithium administration.

## Discussion

We conducted an observational study of lithium in patients with severe steroid refractory intestinal GVHD with a focus on those with extensively denuded mucosa. The study was based on the premise that restoration of mucosal barrier function was a prerequisite to resolution of systemic GVHD in this subset of patients. The primary intent was to evaluate the potential merit of further studies of lithium and to obtain preliminary data for use in the design of a structured next phase study. Due to concerns of safety and lack of preclinical data supporting use of lithium in this context, the study parameters, including timing and duration of lithium administration, were left to the discretion of the primary providers and patients. To minimize the inherent bias in the evaluation of unstructured data, we chose survival to be the main outcome measure to be used as a basis for judging the merits of more formal studies lithium. Similarly we focused on objective and precise baseline parameters including the date of diagnosis of extensively denuded intestinal mucosa (n = 17), interval time to the onset of lithium administration, and date of initiation of salvage therapy for steroid refractory GVHD.

The study data was reviewed for differences between responders and non-responders that might guide study eligibility criteria for a next phase clinical trial. We observed that all eight patients who survived one year had two characteristics in common: administration of lithium was started within 3 days after the endoscopic diagnosis of denuded mucosa and salvage therapy for refractory GVHD was not administered before the onset of lithium administration.

Other measures of GVHD severity which may have contributed to variation in outcomes were not clearly different between survivors and non-survivors, all of whom had a severe clinical course. These measures included volume of diarrhea, severity of abdominal pain, and the incidence of end organ compromise, hypoalbuminemia, and fungal and viral infections, including CMV.

Lithium was administered in an effort to promote mucosal regeneration. However, serial endoscopic evaluations at key time points could not be performed, and therefore, the rate or degree of mucosal recovery could not be adequately assessed.

Serum lithium concentrations were maintained at low levels due to concern for toxicity. These observations raise the question of whether the lithium doses were adequate for optimal evaluation of efficacy. The optimal dose of lithium remains to be determined.

Lithium appeared to be well tolerated, but symptoms attributable to lithium toxicity were difficult to assess in this non-randomized population of critically ill and highly symptomatic patients who have high, > 70%, reported incidence of delirium events [52]. The main reported toxicities were fatigue, dulled sensorium, and flattened affect. Lithium can cause decreased urinary concentrating ability, and this effect should be considered in patients with renal insufficiency. Use of lithium to upregulate a component of Wnt signaling raised concern for recurrence of hematologic malignancy. Three patients had recurrent malignancy. The relapses occurred either very early or very late, and the 15% rate was comparable to the reported 26% rate among 1148 consecutive transplants at the FHCRC during the years 2003–2007 [51]. Nonetheless, the potential effects of lithium on hematologic malignancy remain undefined.

The survival data compare favorably with the historical FHCRC and with other reported survival data. Jamani et al reported less than 20% 2 year survival and less than 10% 5 year survival in a cohort of severe, grade 3–4 (stage 2–4), steroid refractory GVHD and similar median survival in review of post year 2000 published studies [53–55]. A 2 year survival rate of 10% was noted in 69 adults with steroid-refractory stage 3 or 4 intestinal GVHD (including all endoscopic grades) in a cohort of 1447 allogeneic transplants at the FHCRC during the 6 years preceding our study The 27 adults with grade 3 (on scale of 1–3) endoscopic appearance in the duodenum or colon had 0% 1 year survival. The latter included 17 patients whose endoscopy report included the word denuded. Similarly, Abraham et al reported 0% 5 year survival in allogeneic HCT patients with intestinal GVHD and severe endoscopic appearance or very severe histological appearance (mucosal sloughing) [56].

In our study of lithium, patients with severe steroid-refractory intestinal GVHD who started lithium administration within 3 days of diagnosis of extensively denuded mucosa (equivalent to extreme grade 3 endoscopic appearance) had a 67% survival at 1 year and 50% survival at 5 years. This results was similar to the historical survival of the total cohort of transplanted adult patients at the FHCRC in the years 2003–2007 reported by Gooley et al. and better than the expected near 0% survival previously observed in this subset of patients comparable to those enrolled in the current study. Those who started lithium administration within 3 days of diagnosis of denuded mucosa and less than 7 days from salvage therapy for GVHD, had an 80% 1 year survival and 60% 5 year survival. Although the observational study design lacked the statistical power and controls to determine efficacy of lithium, the factual observations appear to support the conduct of a randomized study of lithium in this patient subset.

Based on the above observations, entry criteria for a structured clinical trial of lithium would include patients with severe steroid-refractory GVHD and endoscopically documented denuded mucosa within less than 7 days from the onset of salvage therapy. Administration of lithium would be initiated within 3 days of endoscopic diagnosis and would be continued for at least 19 days (optimally for more than 28 days) in those without limiting toxicity. The 8 patients who met all of these criteria survived for a median of more than 5 years at last follow-up.

Following the completion of our study, the underlying hypotheses were substantiated by two animal experiments demonstrating that induction of Wnt signaling by R-spondin1, and GSK3 inhibition can ameliorate or prevent fatal GVHD [31,50]. In addition, a large body of literature further established the role of Wnt signaling in mucosal regeneration and explored the effect of GSK3 on innate and adaptive immune responses [36–42,44–49,57–61]. Studies of GSK3 inhibition in experimental inflammatory conditions such as colitis, arthritis, and neuroinflammatory disorders remain exploratory but strongly suggest that GSK3 may be a clinically useful therapeutic target [36–39,46–50,57–63]. To our knowledge, this is the first study in humans to target GSK3 and focus on repair of mucosal barrier dysfunction in the pharmacological management of GVHD. The severe clinical course of GVHD in patients with disrupted mucosal barrier supports the need for further research in this field.

## Supporting information

S1 Appendix TRENDS statement checklist. (PDF)

S2 Appendix Protocol. (PDF)

S1 Fig Flow chart of patients with intact intestinal mucosa. (PDF)
