# Supplementary material for: Pilot study of lithium to restore intestinal barrier function in severe graft-versus-host disease
Source: PLoS One. 2017 Aug 17;12(8):e0183284. doi: 10.1371/journal.pone.0183284 (PMC5560707; doi:10.1371/journal.pone.0183284)
Supplement: S2 Appendix — (PDF) [file pone.0183284.s002.pdf]

Fax #9

2080.00

FRED HUTCHINSON CANCER RESEARCH CENTER
UNIVERSITY OF WASHINGTON SCHOOL OF MEDICINE

Current version: 6/12/2006

1. Title of Protocol: A pilot study to evaluate the potential efficacy of lithium carbonate for stimulation of intestinal recovery in patients with acute GVHD.

Investigator	Professional Title	Area Code (206)
Gerwin Huls, PhD, MD	Visiting Physician, FHCRC	288-1118
Gideon Steinbach, MD	Associate Member, FHCRC, Associate Professor of Medicine, UW	288-2275
David Hockenbery, MD	Member, FHCRC, Professor of Medicine, UW	667-4611
George McDonald, MD	Member, FHCRC, Professor of Medicine, UW	667-6932
Jesse Fann, MD, MPH	Research Associate, FHCRC, Associate Professor of Psychiatry and Behavioral Sciences, UW	288-1030
Howie Shulman	Member, FHCRC, Professor of Pathology, UW	288-2047
Paul Martin, MD	Member, FHCRC, Professor of Medicine, UW	667-4798

Biostatistician: Barry Storer, Ph.D. 667-6151

Research Staff: Terry Furlong, R.N. 667-6819

Emergency (24 hour) Phone: (206) 598-8902; **Fax:** (206) 598-4034

2. Introduction

A. Graft-versus-host disease (GVHD). Allogeneic hematopoietic stem cell transplantation (HSCT) is a potentially curative therapy for a variety of malignancies and non-malignant conditions. In addition to the anti-tumor effect of the conditioning regimen, the graft-versus-tumor (GVT) activity of donor T cells is increasingly recognized as an important component of the overall anti-tumor effect of allogeneic HSCT. However, it has been well established that alloreactive T cells also play a critical role in the development of acute graft-versus-host-disease, which remains one of the main complications of an allogeneic HSCT. Although virtually any host tissue is a potential source of alloantigens, acute GVHD develops only in skin, liver, the gastro-intestinal tract, and possibly lung and thymus (1). Acute GVHD is a significant cause of post-transplant morbidity and mortality.

B. Intestinal GVHD. The gut is considered to play a major role in laying in the foundation for the pathologic processes that will result in acute GVHD (2). Acute intestinal GVHD may manifest as nausea, vomiting, diarrhea, ileus, abdominal cramps, pain and bleeding. The latter is a usually a sign of advanced disease, associated with breaks in the mucosal barrier, including erosions, ulcers, or in severe cases, sloughing of the lining epithelium. The hallmark histological manifestation of acute intestinal GVHD is apoptosis in the epithelium lining the crypts, and secondary dropout of cells and crypts. (3-6). In intestinal GVHD the continuous attack of the intestinal epithelium can result in de-epithelialization of the mucosa. In extreme cases denudation of large segments of the intestine is observed and is associated with a grim prognosis (7). In our experience, epithelial regeneration of denuded mucosa is a distinctly unusual outcome in patients with acute GVHD.

In a retrospective review, we identified 74 patients who received secondary therapy for acute GVHD with peak stage 2 – 4 gastrointestinal involvement between 2000 and 2004. Only 39 (53%) of these patients survived for more than 60 days after starting secondary treatment for acute GVHD. Thirty-three of these patients received third-line therapy for acute GVHD, and only 8 (24%) survived beyond 60 days from third-line therapy.

C. Implications of denuded mucosa in patients with GVHD. Extensive denudation of the intestinal mucosa is a grave clinical condition, rarely observed outside the context of GVHD. Loss of the mucosal barrier exposes the patient to infection, a condition that is exacerbated by immunosuppressive treatment of GVHD. In addition, loss of the mucosal barrier subjects the patient to ongoing loss of fluids, electrolytes, proteins and blood, as well as to loss of the capacity to absorb nutrients. The condition is exacerbated by the patient's inability to eat, because foods, and short chain fatty acids derived from bacterial fermentation of dietary carbohydrate, are key stimulants to epithelial regeneration through release of mucosal growth factors and supply of metabolic fuel for enterocytes. Persistent immunological destruction of intestinal stem cells in the presence of GVHD disrupts the process of re-epithelialization that requires functional stem cells. Recovery is contingent both on the control of GVHD and on rapid regeneration of the intestinal mucosa. Currently, no pharmacological agents are available to induce regeneration of the intestinal epithelium. Hence, the condition is generally fatal. Availability of an agent to induce extensive epithelial regeneration could be of significant clinical value.

2080.00

3. Background

A. Wnt signaling. The Wnt family of signaling molecules regulates numerous processes in animal development and has increasingly been implicated in tissue homeostasis in adult organisms (reviewed in 8,9). A hallmark of Wnt pathway activation is the elevation of cytoplasmic β-catenin protein levels. In the absence of Wnt signaling, β-catenin is phosphorylated by the serine/threonine kinases, Casein Kinase and GSK-3. The interaction between these kinases and β-catenin is facilitated by the scaffolding proteins Axin and APC. Together these proteins form the degradation complex, which phosphorylate β-catenin which is subsequently recognized by β-TRCP and targeted for ubiquination, and degraded by the proteasome. Activation of Wnt signaling inhibits β-catenin phosphorylation and hence its degradation. The elevation of β-catenin levels leads to its nuclear accumulation and complex formation with TCF/LEF transcription factors to activate transcription of target genes.

B. Wnt signaling and intestinal epithelium (reviewed in 10-12). The relevance of Wnt signaling to intestinal biology was established more than 10 years ago when it was found that germ-line mutations of the tumor suppressor gene APC was responsible for the development of colonic adenomas in patients with familial adenomatous polyposis (13,14). Subsequently, combined work from several laboratories led to the finding that inactivation of APC in CRC cells results in constitutively active Wnt signaling (15,16). Since these early findings, a much richer picture has emerged. It is now recognized that Wnt signaling not only drives colonic tumorigenesis but is also required at different stages of gut development, as well as during epithelial homeostasis.

The self-renewing capacity of the intestine depends on the existence of stem cells (10-12). Currently, there are several lines of evidence that show that normal proliferation of the transit-amplifying cells is entirely dependent on continual stimulation of the Wnt pathway. First, removal of Tcf4, β-catenin, or over-expression of the Wnt inhibitor Dkk-1 results in a severe loss of proliferative epithelial cells in both the fetal and adult intestine (17-20). Cell cycle arrest is also observed in CRC cell lines in which β-catenin/Tcf activity is blocked either through expression of dominant-negative Tcf4 or knockdown of β-catenin (21,22). Consistent with these results, mutations in the negative regulator of Wnt signaling APC, or over-expression of oncogenic forms of β-catenin result in hyperproliferation of the epithelium (23-26). Lastly, progenitors located at the bottom of the crypts accumulate nuclear β-catenin, implying that these cells respond to Wnt signaling (21). The proliferative effects of Wnt signaling on crypt progenitors have been linked to cell cycle regulators such as c-Myc (27).

Recently it was shown that R-spondin1 activates β-catenin-mediated signaling and dramatically increases the proliferation and growth of the small and large intestines in mice (28). Treatment with R-spondin1 was shown to mitigate the adverse gastrointestinal effects (enteritis, diarrhea, weight loss, and impaired epithelial integrity) associated with the chemotherapeutic agent fluorouracil without increasing tumor growth, as compared with fluorouracil treatment alone. A single dose of R-spondin1 resulted in rapid proliferation of crypt progenitors. These experiments set stage for the use of activators of Wnt signaling to restore the damaged epithelial layer.

C. Effects of lithium on intracellular signaling. Lithium, as simple cation, is the simplest drug in the modern pharmacopoeia (29, 30). Lithium has been shown to be a direct, reversible inhibitor of Glycogen Synthase Kinase 3 (GSK-3) (31). Three closely related forms of GSK3 (GSK-3α, GSK-3β and

GSK-3γ) have a major role in Wnt and Hedgehog signalling pathways and regulate cell-division cycle, stem-cell renewal and differentiation, apoptosis, circadian rhythm, transcription and insulin action. More than 30 inhibitors of GSK-3 have been identified (32). The selectivity of most of the available GSK-3 inhibitors is poorly known and, essentially, based on their evaluation on limited panels of kinases (33). Lithium is a relative weak inhibitor of GSK-3α and GSK-3β (Ki = 2 mM, IC50 = 2000 μM), but has no effect on cyclin dependent kinases reported. In contrast, for example the compound 6-Bromoindirubin-3'-oxime (BIO) has an IC_{50} of 0.005 μM for GSK-3α and GSK-3β but also an IC_{50} of 0.320 μM for CDK1-cyclin B complex. Addition of GSK-3 inhibitors to embryonic stem cells (ESCs) has lead to different outcomes, varying from neuronal differentiation to supporting the renewal of human and mouse ESCs and the maintenance of their pluripotency (34,35). Current interest in GSK-3 has overshadowed a second, well-characterized signal transduction pathway that is subject to lithium inhibition, lowering the cellular concentration of the second-messenger inositol (1,4,5)-triphosphate (36).

D. Potential effects of lithium on regeneration of intestinal epithelium. As discussed, various data show that lithium can activate the Wnt signaling route and Wnt signaling plays an important role in intestinal homeostasis. Treatment of mice with Lithium results in enlarged crypts (Dr. H. Clevers, personal communication). Recently, elegant experiments with R-spondin1 show the power of this drug, presumably mediated via Wnt signaling, to restore damaged intestinal epithelium. Since, lithium can activate Wnt signaling, it is tempting to speculate that lithium might have a role in restoring damaged epithelium.

Current treatments of GVHD focus on the immune mediated attack, with much less attention to interventions that might stimulate restoration of the attacked tissue. One recommended practice is to feed patients with severe intestinal GVHD green bananas, which provide a potent substrate for colonic generation of short chain fatty acids. Besides passive transport into colonocytes, short chain fatty acids have been shown to activate Wnt signaling (37).

Recently, two patients with steroid refractory GVHD and extensively denuded colonic mucosa have been treated with lithium at the FHCRC. Both patients experienced regeneration of the colonic epithelium, even in the presence of ongoing gut GVHD. We hypothesize that activation of Wnt signaling by lithium induced this regeneration of the intestinal epithelial layer, since, in our experience, denuded intestine rarely shows such epithelial regeneration in patients with GVHD.

4. Objectives

A. Primary objective. The primary objective of this pilot study is to evaluate the effects of lithium on functional and mucosal anatomic recovery in the small or large bowel of patients with acute GVHD. Functional recovery will be evaluated according to changes in clinical manifestations of gastrointestinal GVHD. Mucosal anatomic recovery will be evaluated by review of results from clinically indicated endoscopic evaluations.

B. Secondary objective. The secondary objective of this study is to assess the tolerability of lithium administration in allogeneic HCT recipients.

Additional laboratory studies will be performed to examine mechanisms of action of lithium on intestinal cells. Colon biopsies will be studied for accumulation of nuclear β-catenin in epithelial cells, a hallmark of active Wnt signaling. The expansion of proliferating cells in the colon will be studied by staining biopsies with markers of proliferation (such as Ki-67).

5. Patient Selection

A. Inclusions. Patients may be enrolled for either of the following two reasons.

1) Patient with a diagnosis of severe intestinal GVHD that is not improving at any time after initial treatment with glucocorticoids for at least 7 days are eligible for enrollment. Measures indicating severity of GVHD will include a) persistent diarrhea with average daily stool volumes > 500 mL per day or b) persistent hemorrhage that is detectable by visual inspection of the stool.

2) Patients with denuded mucosa caused by GVHD are eligible for enrollment, regardless of prior treatment for acute GVHD. Denuded mucosa is defined as loss (i.e., erosion or sloughing) of the epithelium in a) at least one-third of the surface area in a 30 cm colonic segment (i.e., rectosigmoid, descending or transverse colon) or b) at least one fifth of the surface area of the second portion of the duodenum, as estimated by endoscopic evaluation. Denuded mucosa must be documented by images of the duodenum and colon and by histologic evaluation of the colon.

3) All subjects must provide written informed consent with the use of forms approved by the FHCRC IRB.

B. Exclusions.

1) Significant renal dysfunction (estimated creatinine clearance < 30 mL/min), or
2) Persistent or recurrent malignancy, or
3) Secondary malignancy, or
4) Patients who had autologous or syngeneic marrow transplantation, or
5) Presence of any cause of intestinal symptoms or ulceration other than GVHD, or
6) Patients with any psychological, familial, sociological or geographical condition potentially hampering compliance with the study protocol will be excluded.

6. Evaluation and Counseling of Patient. The protocol will be discussed thoroughly with patient, donor and family, and all known risks to the patient and donor will be described. The procedure and alternative forms of therapy will be presented as objectively as possible and the risks and hazards of the procedure explained to the patient or, in the case of minors, to the patient's responsible family members. Consent will be obtained using forms approved by the Institutional Review Board of the Fred Hutchinson Cancer Research Center. A summary of the conference should be dictated for the medical record detailing what was covered.

7. Protocol Registration. The research nurse will register the patient with the Registration Office, (206) 667-4728, between 8:30 am and 4:00 pm, Monday through Friday. After hours, the Registration Office can be reached by paging (206) 995-7437.

8. Plan of Treatment

A. Drug Administration

Lithium carbonate slow release will be dosed as follows:

Day 1:	Lithium carbonate slow release 450 mg once daily
Day 2:	Lithium carbonate slow release 450 mg bid
Day 3,4,5,6:	Lithium carbonate slow release 450 mg tid
Day 7:	First serum level drawn. Doses will be then be adjusted as needed to maintain serum lithium levels of 0.8 to 1.2 mmol/L.

Treatment of GVHD will be decided by the primary physician and is not affected by the study.

Administration of lithium under this protocol may be continued for up to 8 weeks. Administration of lithium separately from this study may be continued beyond 8 weeks at the discretion of the attending physician.

B. Discontinuation of drug administration. Administration of lithium must be discontinued at the request of the subject or if treatment jeopardizes subject welfare in the judgment of the attending physician or the investigators. Administration of lithium must also be discontinued in patients who develop significant renal dysfunction (estimated creatinine clearance < 30 mL/min) or recurrent or secondary malignancy.

9. Evaluation. Patients will be considered for enrollment in this study only if a prior clinical Gastroentrology consultation confirmed the diagnosis of acute intestinal GVHD and excluded other gastrointestinal complications. Such consultation generally includes esophagogastroduodenoscopy (EGD), colonoscopy or sigmoidoscopy, as clinically indicated. Baseline (pre-treatment) EGD, sigmoidoscopy or colonoscopy is not necessary for enrollment is this study. Clinical assessments of GVHD will be provided according to the standard of care. EGD, colonoscopy or sigmoidoscopy will be repeated as clinically indicated according to the standard of care and are not required by this protocol. The timing of these procedures will be dictated by clinical standards of care.

A. Before treatment.

1) Medical records will be reviewed to determine eligibility for the study.

2) Medical records will be reviewed at baseline to summarize oral intake, administration of parenteral nutrition, daily stool volumes (with estimated correction for urinary mixing, if necessary), presence of abdominal pain or cramps and use of opioid analgesics during the week before enrollment in the study. Records will be annotated to indicate stool consistency (fully formed, partially formed, liquid) and the presence or absence of stool blood detectable by visual inspection of the stool.

3) Medical records will be reviewed to summarize results from previous Gastroenterology consultations and EGD, colonoscopy or sigmoidoscopy.

4) Medical records will be reviewed to summarize all systemic immunosuppressive medications and any orally administered topically active glucocorticoid medications previously given for treatment of acute GVHD.

5) For English-speaking patients, a baseline questionnaire related to adverse events potentially attributable to lithium will be administered at baseline.

6) For English-speaking patients and caregivers, a baseline questionnaire related to mood will be completed by the patient and caregiver.

B. During treatment

1) Medical records will be reviewed at weekly intervals to summarize oral intake, administration of parenteral nutrition, daily stool volumes (with estimated correction for urinary mixing, if necessary), presence of abdominal pain or cramps and use of opioid analgesics. Records will be annotated to indicate stool consistency (fully formed, partially formed, liquid) and the presence or absence of stool blood detectable by visual inspection of the stool.

2) Hematopoietic cell transplant laboratory monitoring will be continued per standard practice. Adverse effects of lithium, including hyperglycemia, electrolyte abnormalities, renal dysfunction and leukocytosis will be monitored within this routine laboratory testing. Study personnel will review laboratory results for possible adverse effects related to the administration of lithium carbonate.

3) Medical records will be reviewed to summarize results from previous Gastroenterology consultations and EGD, colonoscopy or sigmoidoscopy.

4) Medical records will be reviewed to summarize all systemic immunosuppressive medications and any orally administered topically active glucocorticoid medications.

5) Lithium levels will be measured at 1 week after starting treatment and then twice weekly thereafter.

6) For English-speaking patients, a questionnaire related to mood and adverse events potentially attributable to lithium will be administered once weekly throughout the administration of lithium.

7) For English-speaking patients and caregivers, a questionnaire related to mood will be completed once weekly by the patient and caregiver throughout the duration of lithium administration.

C. After treatment.

1) Medical records will be reviewed to summarize late complications attributable to treatment with lithium, changes in systemic immunosuppressive treatment, development of chronic GVHD, recurrent malignancy, and survival.

2) Biopsy specimens that are not needed for clinical care may be used for additional histopathological analysis. For example, sections may be studied for markers of proliferation (e.g., Ki-67) and accumulation of nuclear β-catenin in epithelial cells, a hallmark of Wnt signaling.

10. Pharmacokinetics, Toxicities and Complications

A. Pharmacokinetics. Absorption of Lithium carbonate is complete. Lithium is eliminated almost completely through renal excretion. $T_{1/2}$ is 14 to 24 hours.

B. Side effects (with standard dose). Initially, at therapeutic blood levels, nausea, vomiting, diarrhea, tremor of hands, increased weight, thirst, edema, acne, psoriasis, alopecia, polyuria and polydipsia, goiter and hypothyroidism can occur. Hypothyroidism can be corrected by addition of thyroid hormone. Mild intoxication can result in apathy, dullness, muscle weakness, dysarthria and leukocytopenia. Independent of serum Lithium level, changes in ECG, EEG, nephrogenic diabetes insipidus, hypovolemia related to decreased renal absorption of sodium, skin ulcers on the legs, transient hyperglycemia, leukocytosis, pruritus and metallic taste can occur.

C. Drug Interactions. Indomethacin, piroxicam, phenylbutazone, diclofenac, ibuprofen, naproxen, ACE-inhibitors, low salt diet and thiazide diuretics decrease the renal excretion of Lithium and thereby increase the risk of intoxication. Lithium levels in the blood may be higher when lithium is taken in combination with serotonin reuptake inhibitors (SSRI). Lithium and iodide can work synergistically in causing hypothyroidism. Sodium containing preparations, acetazolamide and theophylline can increase

lithium excretion. Combination of lithium with high doses of haldol can cause neurotoxicity and extra-pyramidal signs (rarely permanent). In combination with verapamil and diltiazem lithium can cause neurotoxicity. Combined with MAO-inhibitors, lithium can cause "serotonergic-syndrome".

11. Protocol Enrollment and Special Considerations

Projected Target Accrual
ETHNIC AND GENDER DISTRIBUTION CHART

TARGETED / PLANNED ENROLLMENT: Number of Subjects			
Ethnic Category	Sex / Gender		
	**Females**	**Males**	**Total**
Hispanic or Latino	0	1	1
Not Hispanic or Latino	9	15	24
Ethnic Category Total of All Subjects*	9	16	25
**Racial Categories**			
American Indian / Alaska Native	0	0	0
Asian	0	1	1
Native Hawaiian or Other Pacific Islander	0	0	0
Black or African American	0	0	0
White	9	15	24
Racial Categories: Total of All Subjects*	9	16	25

12. Guidelines for Serious Adverse Event Reporting

The following guidelines are the minimum serious adverse event (SAE) reporting guidelines for Category 1 and 2 studies conducted at the Fred Hutchinson Cancer Research Center. In some circumstances the protocol may have additional reporting requirements that should be followed for adverse event reporting.

Expedited Reporting Requirements:

All unexpected and serious adverse events which may be due to study treatment or intervention must be reported to the FHCRC Institutional Review Office as soon as possible but within at least 7 calendar days of the investigator learning of the event.

Definitions:

Adverse Event - Any untoward medical occurrence in a patient or clinical investigation subject administered a pharmaceutical product, medical treatment or procedure and which does not necessarily

have to have a causal relationship with this treatment. An adverse event can therefore be any unfavorable and unintended sign (including an abnormal laboratory finding, for example), symptom, or disease temporally associated with the use of a medicinal product, medical treatment or procedure whether or not considered related to the medicinal product.

Life-threatening Adverse Event – Any adverse event that places the patient or subject, in view of the investigator, at immediate risk of death from the reaction.

Unexpected Adverse Event – An adverse event, the nature or severity of which is not consistent with the applicable product information (e.g., Investigator's Brochure for an unapproved investigational product or package insert/summary of product characteristics for an approved product). If applicable product information is not available, such as for studies that do not involve pharmaceutical products or devices, an unexpected adverse event is an adverse event that was not described in the study protocol or informed consent.

Serious Adverse Event (SAE) – Any adverse event occurring that results in any of the following outcomes:

- death;
- a life-threatening adverse event (real risk of dying);
- inpatient hospitalization or prolongation of existing hospitalization;
- a persistent or significant disability/incapacity;
- a congenital anomaly;
- requires intervention to prevent permanent impairment of damage.

To ensure no confusion or misunderstanding exist of the differences between the terms "serious" and "severe," which are not synonymous the following note of clarification is provided:

> ***The term "severe" is often used to describe the intensity (severity) or a specific event (as in mild, moderate or severe myocardial infarction); the event itself, however, may be of relatively minor medical significance (such as severe headache). This is*** **not** ***the same as "serious," which is based on patient/event*** **outcome or action** ***criteria usually associated with events that pose a threat to a patient's life or functioning. Seriousness (not severity) serves as a guide for defining regulatory obligations.***

Attribution - The FHCRC designation for the determination of whether an adverse event is related to a medical product, treatment or procedure will be as follows:

- Related – includes adverse events that are definitely, probably, or possibly related to the medical treatment or procedure;
- Not Related – includes adverse events are doubtfully related or clearly not related to the medical treatment or procedure.

The FHCRC Serious Adverse Event (SAE) Report Form should be completed for all adverse events that meet the expedited reporting requirements. The SAE form should be faxed to the IRO at (206) 667-6831. All available information should be submitted but it is acceptable to fax an incomplete report form at the initial report. A completed report should be faxed as soon as possible but must be received within 15 calendar days.

Serious adverse events that do not meet the requirement for expedited reporting (not related to study treatment or expected) must be reported to the IRB as part of the annual renewal of the protocol.

13. **Records.** Clinical Statistics maintains a patient database at FHCRC to allow storage and retrieval of patient data collected from a wide variety of sources. The investigator will ensure that data collected conform to all established guidelines for coding, collection, key entry and verification. Each patient is assigned a unique patient number to assure patient confidentiality. Patients will not be referred to by this number, by name, or by any other individual identifier in any publication or external presentation. The licensed medical records department, affiliated with the institution where the patient receives medical care, maintains all original inpatient and outpatient chart documents. Patient research files are kept in a locked room. They are maintained by the FHCRC data collection staff which is supervised by an A.R.T. Access is restricted to personnel authorized by the Division of Clinical Research.

14. Statistical Considerations

A. Definitions of outcomes and endpoints. A transplant oncologist will be responsible for assessment of GVHD. A gastroenterologist will be responsible for evaluating the endoscopic extent of mucosal denudation. A pathologist will be responsible for the histological grading of biopsies.

1) Functional recovery: Patients who have been treated for at least 4 weeks will be assigned a clinical response category (complete response, partial response, progression) according to the following definitions.

a) Complete clinical response: Absence of any symptoms referable to intestinal graft-versus-host disease.

b) Partial clinical response (38):
   i) resolution of diarrhea or decrease in the three day average stool volume by ≥ 500 ml with clearing of cramps (or withdrawal of narcotic requirements in patients treated for abdominal pain) and grossly visible bleeding if present, OR
   ii) clearing of any cramps (or withdrawal of narcotic requirements in patients treated for abdominal pain) and grossly visible bleeding in patients with diarrhea volumes < 500ml
   iii) stool volumes ≥ 500 mL cannot be evaluated if extensive urinary mixing is present

c) Clinical Progression (38):
   i) Increase in the three day average stool volume by ≥ 500 ml, OR
   ii) the development of new cramps (or new opioid requirement for abdominal pain) or new bleeding

2) Mucosal anatomic recovery: Endoscopic evaluations will be used to assess efficacy, but the frequency and timing of procedures will be dictated by clinical standards of care and not by this protocol. Photographs from EGD, colonoscopy or sigmoidoscopy will be reviewed by a gastroenterologist who was not involved in any of the procedures. Date-blinded photographs from each

anatomic site will be arranged in decreasing order of lesion severity, and differences from one to the next will be graded as no change, marginally improved, definitely improved or healed. Grading assessments will remain unchanged if dates across comparisons are in chronological order. Grading assignments will be changed if dates across comparisons are in reverse chronological order (i.e., marginally or definitely improved will become marginally or definitely worse, and healed will become "new lesion").

Similarly, date-blinded biopsy specimens of the colon will be reviewed by a pathologist who was not involved in the clinical assessment of clinical specimens. Sections will be arranged in decreasing order of lesion severity, and differences will be graded as no change, marginally improved, definitely improved or healed. Grading assessments will remain unchanged if dates across comparisons are in chronological order. Grading assignments will be changed if dates across comparisons are in reverse chronological order (i.e., marginally or definitely improved will become marginally or definitely worse, and healed will become "new lesion").

3) Tolerability of lithium: Grade III and IV adverse events attributable to treatment with lithium will be monitored on a continuing basis and their frequencies will be reported semi-annually. Adverse events will be categorized using the NCI Common Terminology Criteria for Adverse Events, Version 2.0.

B. Statistical design. This is a single-center, open-label pilot study to study the efficacy and tolerability of lithium carbonate in combination with immunosuppressive therapy for GVHD of the intestinal tract. The estimated accrual rate for this study is approximately 2 – 3 patients per month.

C. Statistical Analysis. Results will be analyzed descriptively in terms of the proportions of partial and complete responses and the frequencies of adverse events. In evaluating the potential merit of further studies with the use of lithium in patients with acute GVHD of the intestinal tract, particular attention will be given to subjects with denuded mucosa, in whom mucosal regeneration can be evaluated by comparing results of sequential endoscopic evaluations, since in our previous experience, regeneration of denuded mucosa is a distinctly unusual outcome among patients with acute GVHD. In addition to the endpoints listed above, chronic GVHD, recurrent or progressive malignancy, non-relapse mortality, survival, and relapse/progression-free survival will be summarized as additional outcome measures in this pilot study.

We will evaluate the proportion of patients who survive for more than 60 days from secondary or tertiary treatment of acute GVHD. Historical results indicate 53% survival for more than 60 days from secondary therapy and 24% survival for more than 60 days from tertiary therapy among patients with stage 2 – 4 gastrointestinal GVHD. Results from phase II studies can be heavily influenced by patient selection. For this reason, no formal statistical comparison is planned between patients enrolled in the current pilot study and historical results, but benchmarks of 50% survival for more than 60 days from secondary therapy and 25% survival for more than 60 days from tertiary therapy will be used informally as a basis for judging the merits of more formal studies to evaluate the efficacy of lithium for stimulation of intestinal recovery in patients with acute GVHD.

2080.00

15. Termination of the Study. Up to 25 patients will be enrolled. If none of the initial 6 patients shows complete or partial functional or mucosal anatomic interim recovery at 4 weeks after starting treatment with lithium, the study will be terminated.

16. References

1. Ferrara, J.L., and Deeg, H.J. Graft-versus-host disease. N.Engl.J.Med. 1991;324:667-674.
2. Hill, G.R., and Ferrara, J.L. The primacy of the gastrointestinal tract as a target organ of acute graft-versus-host disease: rationale for the use of cytokine shields in allogeneic bone marrow transplantation. Blood. 2000;95:2754-2759.
3. Epstein, R.J.J., et al. The diagnostic accuracy of the rectal biopsy in acute graft-versus-host disease: A prospective study of 13 patients. Gastroenterology. 1980;78:764-771.
4. Bombi, J.A., et al. Pathology of bone marrow transplantation. Semin.Diagn.Pathol. 1992;9:220-231.
5. Sale, G.E., et al. Gastrointestinal graft-versus-host disease in man: A clinicopathologic study of the rectal biopsy. Am.J.Surg.Pathol. 1979;3:291-299.
6. Shidham, V.B., et al. Colon biopsies for evaluation of acute graft-verus-host disease (A-GVHD) in allogeneic bone marrow transplant patients. BMC Gastroenterology. 2003;3:5-13.
7. Fox, R.J., et al. Denuded bowel after recovery from graft-versus-host disease. Transplantation. 1996;62:1681-1684.
8. Bienz, M., and Clevers, H. Linking colorectal cancer to Wnt signaling. Cell. 2000;103:311-320.
9. Cadigan, K.M., and Nusse, R. Wnt signaling: a common theme in animal development. Genes Dev. 1997;11:3286-3305.
10. Gregorieff, A., and Clevers, H. Wnt signaling in the intestinal epithelium: from endoderm to cancer. Genes Dev. 2005;19:877-890.
11. Radtke, F, and Clevers, H. Self-renewal and cancer of the gut: two sides of a coin. Science. 2005;307:1904-1909.
12. Sancho, E., et al. Signaling pathways in intestinal development and cancer. Annu Rev Cell Dev Biol. 2004;20:695-723.
13. Kinzler, K.W., et al. Identification of FAP locus genes from chromosome 5q21. Science. 1991;253-661-665.
14. Groden, J., et al. Identification and characterization of the familial adenomatous polyposis coli gene. Cell. 1991;66:589-600.
15. Korinek, V., et al. Constitutive transcriptional activation by a beta-catenin-complex in APC-/- colon carcinoma. Science. 1997;275:1784-1787.
16. Morin, P.J., et al. Activation of beta-catenin-Tcf signalling in colon cancer by mutationsin beta-catenin or APC. Science. 1997;275:1787-1790.
17. Korinek, V., et al. Depletion of epithelial stem-cell compartments in the small intestine of mice lacking Tcf-4. Nat.Genet. 1998;19:379-383.
18. Pinto, D., et al. Canonical Wnt signals are essential for homeostasis of the intestinal epithelium. Genes Dev. 2003;17:1709-1713.
19. Ireland, H., et al. Inducible Cre-mediated control of gene expression in the murine gastrointestinal tract: effect of loss of beta-catenin. 2004;126:1236-1246.

20. Kuhnert, F., et al. Essential requirement for Wnt signalling in proliferation of adult small intestine and colon revealed by adenoviral expression of Dickkopf-1. Proc.Natl.Acad.Sci.USA. 2004;101:266-271.
21. Van de Wetering, M., et al. The beta-catenin/TCF-4 complex imposes a crypt progenitor phenotype on colorectal cancer cells. Cell. 2002;111:241-250.
22. Van de Wetering, M., et al. Specific inhibition of gene expression using a stably integrated, inducible small-interfering-RNA vector. EMBO Rep. 2003;4:609-615.
23. Nagase, H., and Nakamura, Y. Mutations of the APC (adenomatous polyposis coli) gene. Hum.Mutat. 1993;2:425-434.
24. Oshima, M., et al. Loss of Apc heterozygosity and abnormal tissue building in nascent intestinal polyps in mice carrying a truncated APC gene. Proc.Natl.Acad.Sci.USA. 1995;92:4482-4486.
25. Kinzler, K.W., and Vogelstein, B. Lessons from hereditary colorectal cancer. Cell. 1996;87:159-170.
26. Sanson, O.J., et al. Loss of Apc in vivo immediately perturbs Wnt signalling, differentiation, and migration. Genes Dev. 2004;18:1385-1390.
27. He, T.C., et al. Identification of c-Myc as a target of the APC pathway. Science. 1998;281:1509-1512.
28. Kim, K-A., et al. Mitogenic influence of human R-Spondin1 on the intestinal epithelium. Science. 2005;309:1256-1259.
29. Jope, R.S. Anti-bipolar therapy: mechanism of action of lithium. Mol. Psychiatry. 1999:4:117-128.
30. Phiel, C.J. and Klein, P.S. Molecular targets of lithium action. Annu.Rev.Pharmacol.Toxicol. 2001;41:789-813.
31. Klein, P.S. and Melton, D.A. A molecular mechanism for the effect of lithium on development. Proc.Natl.Acad.Sci.USA. 1996;93:8455-8459.
32. Meijer, L., Flajolet, M., and Greengard, P. Pharmacological inhibitors of glycogen synthase kinase 3. Trends in Pharmacological Sciences. 2004;25:471-480.
33. Bain, J., et al. The specificities of protein kinase inhibitors: an update. Biochem.J. 2003;371:199-204.
34. Ding, S., et al. Synthetic small molecules that control stem cell fate. Proc.Natl.Acad.Sci.USA. 2003;100:7632-7637.
35. Sato, N., et al. Maintenance of pluripotency in human and mouse embryonic stem cell through activation of Wnt signalling by a pharmacological GSK-3 specific inhibitor. Nat.Med. 2004;10:55-63.
36. Berridge, M.J., et al. Neural and developmental actions of lithium: a unifying hypothesis. Cell. 1989;59:411-419.
37. Bordonaro, M., et al. Cell type- and promoter-dependent modulation of the Wnt signaling pathway by sodium butyrate. Int.J.Cancer. 2002;97:42-51.
38. Martin, P.J., et al. A retrospective analysis of therapy for acute graft-verus-host disease: initial treatment. Blood. 1990;76:1464-1472.

2080.00

Side Effects Checklist

For Severity:
0 = None
1 = Mild (awareness of event but easily tolerated)
2 = Moderate (enough discomfort to cause some interference with usual activities)
3 = Severe (event is incapacitating so that subject is unable to work or do usual activities)
4 = Life-threatening
5 = Fatal

**Side effect**		**Evaluation Day**							
Thirst	Baseline	Day 7	Day 14	Day 21	Day 28	Day 35	Day 42	Day 49	Day 56
Excessive urination									
Edema									
Rash									
Hair loss									
Itching									
Diarrhea / Loose Stools									
Nausea									
Vomiting									
Fatigue / Lack of energy									
Agitation / Restlessness									
Anxiety / Nervousness									
Irritability									
Insomnia									
Coma									
Ataxia or clumsiness									
Seizures									
Confusion									
Slurred speech									
Apathy									
Tremor / Shakiness									
Other									

2080.00

Profile of Mood States (POMS)

Below is a list of words that describe feelings people have. Please circle one number to the right that best describes how you have been feeling during the **PAST 24 HOURS**.

		Not At All	*A Little Bit*	*Moderately*	*Quite A Bit*	*Extremely*
1	**Active**	0	1	2	3	4
2	**Tense**	0	1	2	3	4
3	**Angry**	0	1	2	3	4
4	**Worn out**	0	1	2	3	4
5	**Lively**	0	1	2	3	4
6	**Confused**	0	1	2	3	4
7	**Shaky**	0	1	2	3	4
8	**Sad**	0	1	2	3	4
9	**Grouchy**	0	1	2	3	4
10	**Energetic**	0	1	2	3	4
11	**Unworthy**	0	1	2	3	4
12	**Uneasy**	0	1	2	3	4
13	**Fatigued**	0	1	2	3	4
14	**Annoyed**	0	1	2	3	4
15	**Discouraged**	0	1	2	3	4
16	**Nervous**	0	1	2	3	4
17	**Lonely**	0	1	2	3	4
18	**Muddled**	0	1	2	3	4
19	**Exhausted**	0	1	2	3	4
20	**Anxious**	0	1	2	3	4
21	**Gloomy**	0	1	2	3	4
22	**Sluggish**	0	1	2	3	4
23	**Weary**	0	1	2	3	4
24	**Bewildered**	0	1	2	3	4
25	**Furious**	0	1	2	3	4
26	**Efficient**	0	1	2	3	4
27	**Full of pep**	0	1	2	3	4
28	**Bad-tempered**	0	1	2	3	4
29	**Forgetful**	0	1	2	3	4
30	**Vigorous**	0	1	2	3	4
