# Supplementary figures and images for: Pilot study of lithium to restore intestinal barrier function in severe graft-versus-host disease
Source: PLoS One. 2017 Aug 17;12(8):e0183284. doi: 10.1371/journal.pone.0183284 (PMC5560707; doi:10.1371/journal.pone.0183284)

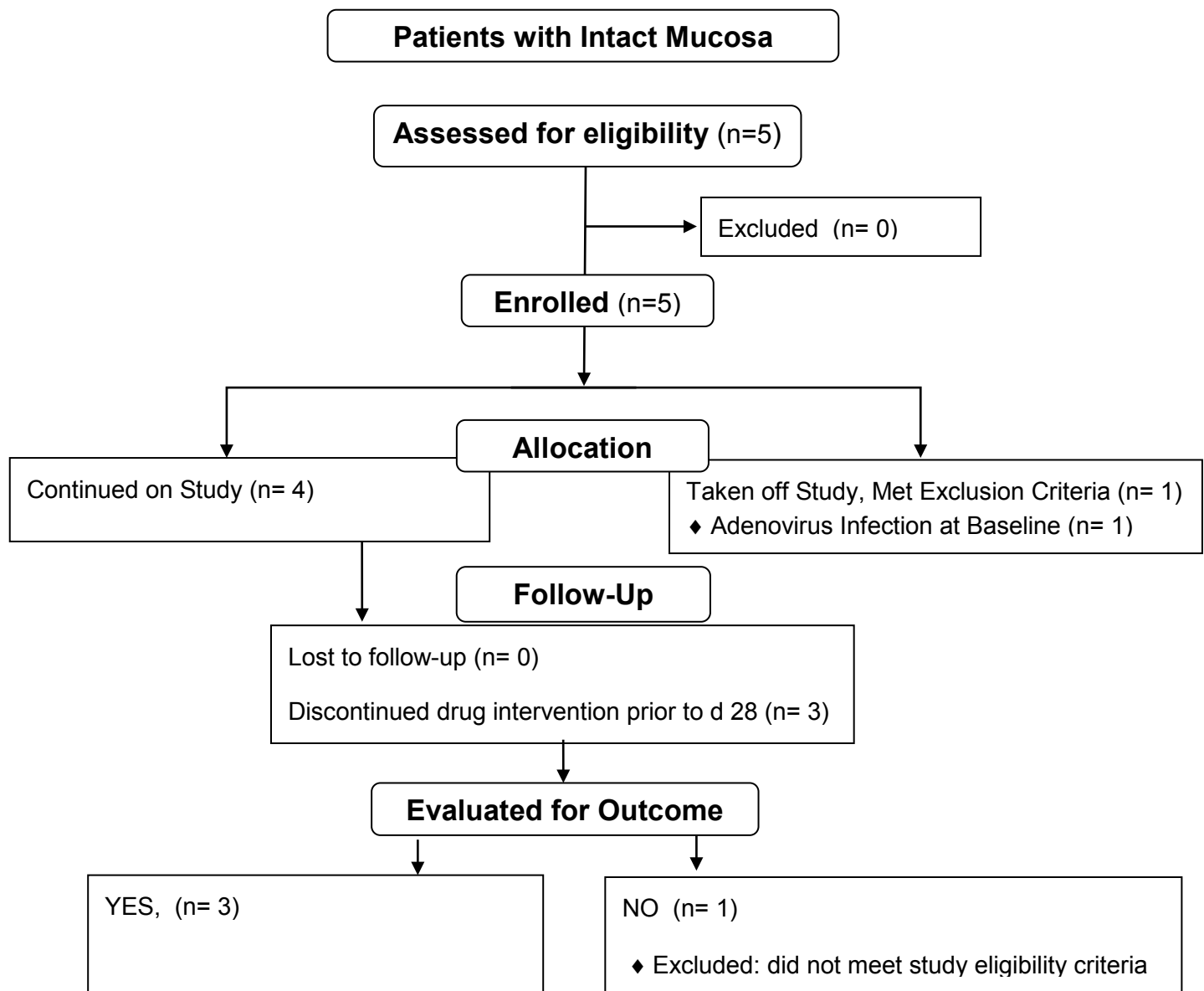

Supplement: S1 Fig — (PDF) [file pone.0183284.s003.pdf]
